# Aurora A site specific TACC3 phosphorylation regulates astral microtubule assembly by stabilizing γ-tubulin ring complex

**DOI:** 10.1186/s12860-019-0242-z

**Published:** 2019-12-10

**Authors:** Resmi Rajeev, Puja Singh, Ananya Asmita, Ushma Anand, Tapas K. Manna

**Affiliations:** 1 School of Biology, Indian Institute of Science Education and Research Thiruvananthapuram, Vithura, Thiruvananthapuram 695551 India; 2 Present Address: Centre for Cellular and Molecular Biology, Uppal Rd, Hyderabad, 500007 India

**Keywords:** Centrosome, Microtubule, Mitosis, Protein phosphorylation, Mitotic spindle

## Abstract

**Background:**

Astral microtubules emanating from the mitotic centrosomes play pivotal roles in defining cell division axis and tissue morphogenesis. Previous studies have demonstrated that human transforming acidic coiled-coil 3 (TACC3), the most conserved TACC family protein, regulates formation of astral microtubules at centrosomes in vertebrate cells by affecting γ-tubulin ring complex (γ-TuRC) assembly. However, the molecular mechanisms underlying such function were not completely understood.

**Results:**

Here, we show that Aurora A site-specific phosphorylation in TACC3 regulates formation of astral microtubules by stabilizing γ-TuRC assembly in human cells. Mutation of the most conserved Aurora A targeting site, Ser 558 to alanine (S558A) in TACC3 results in robust loss of astral microtubules and disrupts localization of the γ-tubulin ring complex (γ-TuRC) proteins at the spindle poles. Under similar condition, phospho-mimicking S558D mutation retains astral microtubules and the γ-TuRC proteins in a manner similar to control cells expressed with wild type TACC3. Time-lapse imaging reveals that S558A mutation leads to defects in positioning of the spindle-poles and thereby causes delay in metaphase to anaphase transition. Biochemical results determine that the Ser 558- phosphorylated TACC3 interacts with the γ-TuRC proteins and further, S558A mutation impairs the interaction. We further reveal that the mutation affects the assembly of γ-TuRC from the small complex components.

**Conclusions:**

The results demonstrate that TACC3 phosphorylation stabilizes γ- tubulin ring complex assembly and thereby regulates formation of centrosomal asters. They also implicate a potential role of TACC3 phosphorylation in the functional integrity of centrosomes/spindle poles.

## Background

High fidelity of chromosome segregation requires proper construction of the mitotic spindles during mitosis. In most animal cells, centrosomes, which are made up of centrioles surrounded by pericentriolar material (PCM), orchestrate the assembly and organization of the mitotic spindles by constituting the microtubule-organizing centers (MTOC). Centrosome maturation occurs during G2/M transition and the process involves recruitment of many structural and regulatory proteins to the PCM [1]. Critical among those are γ-tubulin and its associated protein complexes that are essential for microtubule nucleation from the centrosomes. Specifically, γ-tubulin and conserved γ-tubulin-binding proteins, GCP2 and GCP3 form a sub-complex called γ-tubulin-small complex (γ-TuSC). Multiple such γ-TuSCs together with additional GCP proteins, such as GCP 4, 5 and 6, assemble to constitute the larger γ-tubulin ring complex (γ-TuRC), which serves as the main microtubule-nucleating machinery in cells [2–4]. Centrosomal recruitment of γ-tubulin increases ~ 3–5-folds as cells enter M phase [5] and such robust increase of γ-tubulin seems to be coincident with the increased density of microtubules at the centrosomes [6, 7]. Although the γ-TuRCs are primarily involved in microtubule nucleation, other factors are required for optimal regulation of the function [8–11]. Molecular details of centrosomal microtubule regulation are incompletely understood.

Aurora A is a key centrosomal protein kinase that is essential for mitosis and is widely implicated in human cancers [12]. Aurora A controls mitotic spindle assembly by phosphorylating a number of centrosome- and spindle-associated factors. One such factor is the member of the transforming acidic coiled-coil (TACC) family protein, TACC3. TACC3 is essential in mammals and plays central role in microtubule stability during mitosis. It displays frequent mutations and amplifications in many cancers. Oncogenic fusion products of TACC3 have recently been identified in glioblastoma, bladder, lung and nasopharyngeal carcinomas [13]. Studies in *C. elegans* embryo, *Xenopus* eggs and *Drosophila* neuroblasts have demonstrated that Aurora A activity is essential for localizing TACC3 to the centrosomes [14–17]. Consistently, in human cells, depletion or pharmacological inhibition of Aurora A has been shown to disrupt centrosomal localization of TACC3 [18]. More interestingly, cellular defects caused by Aurora A abrogation display remarkable similarities with TACC3 depletion-induced phenotypes. For example, Aurora A mutation/depletion causes astral microtubule assembly defects and loss of γ- tubulin ring complex (γ-TuRC) recruitment to the centrosomes [16, 19, 20]. Similarly, Maskin/TACC3 immuno-depletion in *Xenopus levies* or siRNA-mediated TACC3 depletion in human cells results in loss of centrosomal asters [9, 21]. Studies in human cells showed that TACC3 is required for localizing the components of the γ-TuRC to the centrosomes in mitotic cells [9]. Moreover, loss of TACC3 affects the assembly of γ-TuRCs from the γ-tubulin small complex components (γ-TuSCs) in human cell lysates, indicating that TACC3 is involved in stabilizing the ring complex [9]. However, the molecular determinants responsible for TACC3-mediated γ-TuRC stabilization are yet to be identified. Furthermore, how this function is linked to astral microtubule regulation at the centrosome remains to be determined.

TACC3 is phosphorylated by Aurora A at three distinct sites, Ser 34, Ser 552, and Ser 558 [22], out of which Ser 558 is most conserved from *Drosophila* to humans. Phospho-deficient mutation of D-TACC at Ser 863 (equivalent site of Ser 558 of human TACC3) in *Drosophila* embryo has been shown to cause astral microtubule defects and embryonic lethality [23]. The mutation affects embryo viability quite drastically. Similar role of Aurora A-mediated phosphorylation has been shown in *Xenopus* TACC3/Maskin [24]. In human cells, loss of astral microtubules induced by TACC3 depletion has been shown to be rescued by TACC3 C-terminal (500–838) region, which consists of the Aurora A-targeting conserved phosphorylation site Ser 558 [9]. Localization of TACC3 to the mitotic centrosomes is also dependent on its phosphorylation at Ser 558 by Aurora A [18, 25]. Although, previous studies in fly and *Xenopus* indicated involvement of TACC3 phosphorylation in astral microtubule formation, mechanism underlying the process was poorly understood. As TACC3 is essential for centrosomal localization of the γ-TuRC and its assembly [9] and it is recruited to the centrosome in its phosphorylated form, we hypothesized that TACC3 phosphorylation may regulate the assembly and stability of γ-TuRC at the centrosome and such activity may be essential for astral microtubule assembly at the centrosomes.

In this study, we have investigated the role of TACC3 phosphorylation in regulation of the γ-tubulin ring complexes and sought to determine its molecular link with astral microtubule assembly at the centrosomes in human cells. By expressing a phospho-deficient Serine to Alanine TACC3 mutant in cells, we have shown that phosphorylation at Ser 558 is essential for proper recruitment of the γ-TuRC proteins to the centrosomes and astral microtubule formation. Live cell imaging has revealed that the phospho-deficient mutation induces characteristic defects in positioning of the mitotic spindle-poles, thereby causing delay in metaphase to anaphase transition and the overall mitosis progression. In contrast, expression of a phospho-mimicking form of TACC3 can restore integration of the γ-TuRC proteins to the mitotic centrosomes, and further efficiently rescues the loss of astral microtubules. We further show that the Ser 558-phosphorylated TACC3 remains associated with the γ-TuRC and further have demonstrated that the phosphorylation plays essential role in TACC3 interaction with γ-TuRC and stabilizing the assembly of the γ-TuRC complex from the γ-tubulin small complex components. Our results uncover a hitherto unknown function of TACC3 phosphorylation in regulating the assembly of γ-TuRC and its integration to the mitotic centrosomes. They also indicate that the phospho-TACC3-mediated γ-TuRC stabilization and integration to the mitotic centrosomes regulate astral microtubule assembly.

## Results

### Phosphorylated TACC3 is required for formation of astral microtubules at the centrosomes/spindle poles

**Fig. 1:**
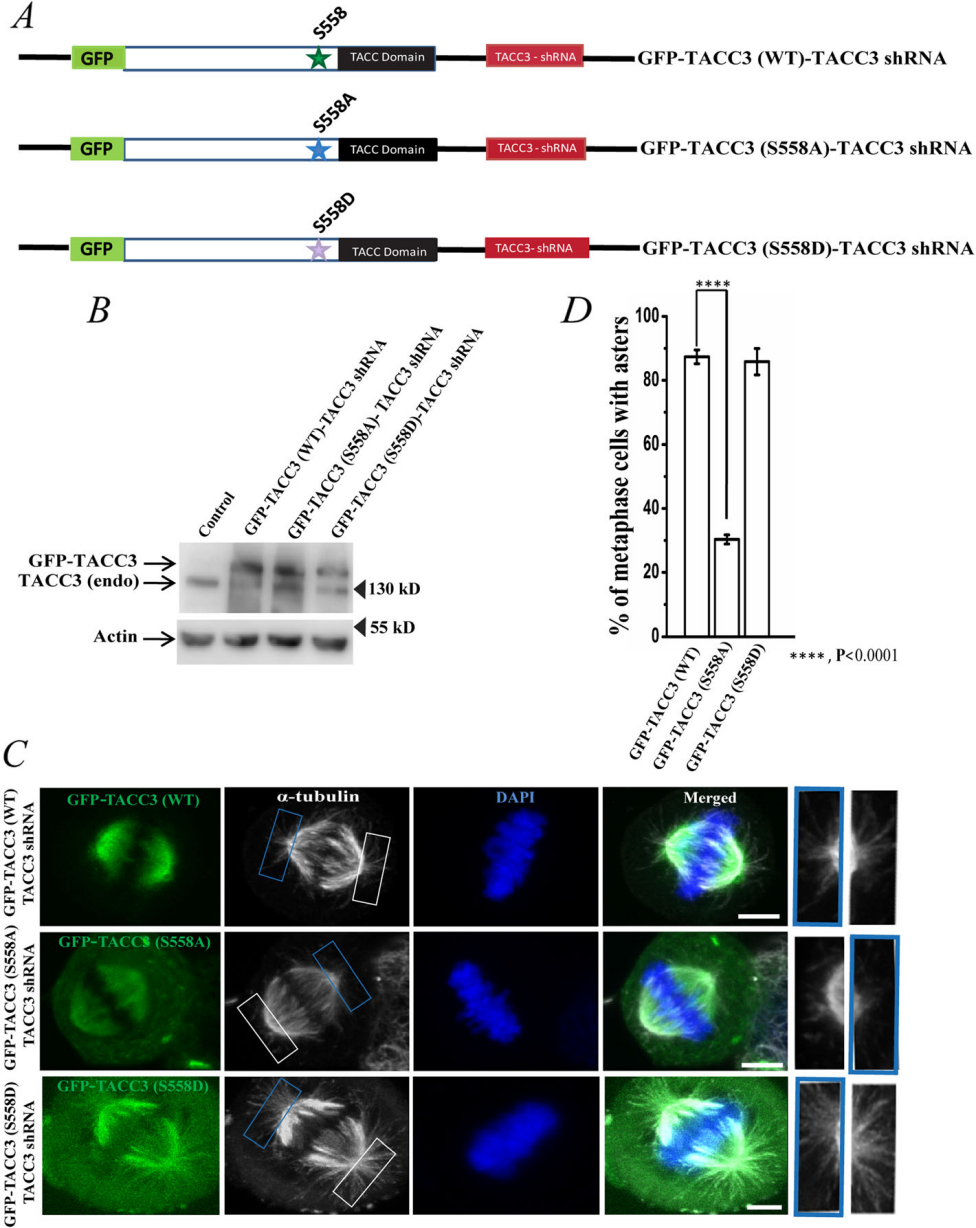
Phosphorylation of TACC3 at Ser 558 is involved in astral microtubule assembly at the centrosomes. **a***.* Schematic representation of GFP-TACC3 (WT)-TACC3 shRNA, GFP-TACC3 (S558A)-TACC3 shRNA and the GFP-TACC3 (S558D)-TACC3 shRNA constructs. **b***.* Lysates of HeLa cells transfected with GFP-TACC3 (WT)-TACC3 shRNA, GFP-TACC3 (S558A)-TACC3 shRNA and the GFP-TACC3 (S558D)-TACC3 shRNA for 48 h were analyzed by Western blot to detect the levels of exogenous TACC3 proteins with simultaneous depletion of endogenous TACC3. Both endogenous TACC3 and the exogenous TACC3 proteins were probed with mouse monoclonal anti-TACC3 antibody. Actin was probed as control. **c***.* Representative confocal images of GFP-TACC3 (WT)-TACC3 shRNA, GFP-TACC3 (S558A)-TACC3 shRNA or GFP-TACC3 (S558D)-TACC3 shRNA transfected mitotic HeLa Kyoto cells showing the differences in astral microtubules. Scale bar, 5 μm, Microtubules (red channel shown in grey) were stained with mouse monoclonal anti-α-tubulin antibody. The arrows show centrosomes/spindle poles. Centrosomal regions are also shown in enlarged view. **d***.* Bar graph shows the quantification of metaphase cells with astral microtubule assembly defects in different conditions as of C in HeLa Kyoto cells. Percentage refers to the ratio of the number of metaphase cells with loss of asters and the total number of metaphase cells. The bars represent mean +/− S.E. Number of mitotic cells counted = 50 each (three independent experiments)

We first determined whether phosphorylation at Ser 558 site of human TACC3 imparts any role in the TACC3-mediated microtubule assembly at the centrosomes in mitotic cells. HeLa Kyoto cells were transiently transfected with a fusion plasmid consisting of a shRNA-resistant phospho-deficient (S558A) TACC3 and TACC3 shRNA, hereafter referred as GFP-TACC3 (S558A)-TACC3 shRNA. Transfection of the plasmids containing the phospho-mimicking TACC3 (S558D) mutant and TACC3 shRNA, referred as GFP-TACC3 (S558D)-TACC3 shRNA or wild-type TACC3 and TACC3 shRNA, GFP-TACC3 (WT)-TACC3 shRNA were also performed in parallel for comparison (Fig. 1a). Transfection of the constructs allowed expression of TACC3 (S558A) or TACC3 (S558D) or the TACC3 (WT) with simultaneous depletion of the endogenous TACC3 protein by TACC3 shRNA present in the constructs (Fig. 1b) [26]. Mitotic synchronized metaphase cells expressing the TACC3 S558A mutant showed robust loss of astral microtubules as compared to the TACC3 (WT)-expressed cells (Fig. 1c). Cells expressed with the TACC3 (S558D) mutant showed normal morphology of astral microtubules like the WT cells. While ~ 90% TACC3 WT- expressed metaphase cells had normal astral microtubules around the spindle poles, S558A mutation led to loss of asters in ~ 60–70% metaphase cells. Only ~ 20% cells showed nearly normal astral microtubule morphology (Fig. 1d). Further, expression of the S558D mutant rescued the phenotype similar to control (TACC3 WT-expressed) cells or in some cases relatively more denser asters than the WT cells (bottom panel of Fig. 1c, plot in 1D). Therefore, consistent with the results in *Drosophila* and *Xenopus* models, our results in human cells support that phosphorylation at Ser 558 site in TACC3 is essential for formation of mitotic asters.

**Fig. 2:**
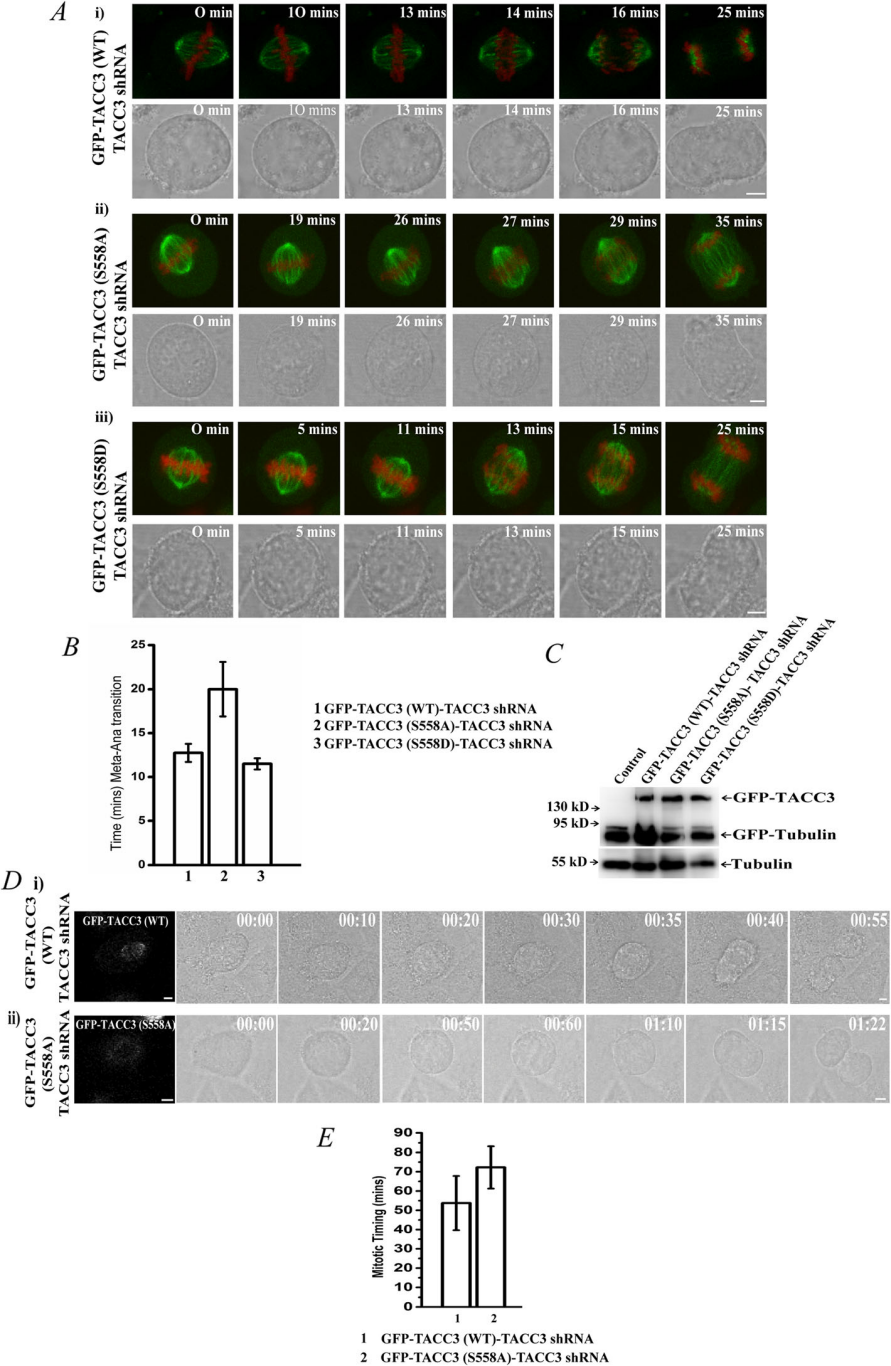
S558A mutation in TACC3 affects correct positioning of mitotic centrosomes. **a***.* Time-lapse images of live metaphase-synchronized HeLa Kyoto cells stably expressed with α-tubulin-GFP and H2B-mCherry were transfected with i) GFP-TACC3 (WT)-TACC3 shRNA, ii) GFP-TACC3 (S558A)-TACC3 shRNA or iii) GFP-TACC3 (S558D)-TACC3 shRNA are shown. The time at which chromosomes were visibly aligned to the metaphase plate (based on the H2B mCherry staining) after thymidine release refers to t = 0 min. GFP and bright-field images of the same cell are shown in top and bottom panel, respectively in each. Respective time-lapse movies are shown in Additional file 1: Movie S1, Additional file 2: Movie S2 and Additional file 3: Movie S3. **b***.* Times of metaphase to anaphase progression in TACC3 WT, S558A and S558D expressed mitotic cells are plotted. Data are mean +/− S. E. (three experiments) **c***.* Since the cells imaged in A are also expressed with α-tubulin-GFP, the expressions of the GFP-tagged TACC3 variants were confirmed by Western blot. GFP-TACC3 constructs (WT or the mutants) were expressed in all the three conditions. **d***.* The bright-field time lapse images of HeLa Kyoto cells transfected with i) GFP-TACC3 (WT)-TACC3 shRNA, or ii) GFP-TACC3 (S558A)-TACC3 shRNA followed by thymidine treatment and release thereafter, were imaged soon after the cells entered mitosis after thymidine release (t = 00:00 h:min). The images of the same mitotic cells from the start to end of mitosis are shown. It was confirmed by snapshot GFP imaging (at t = 0) that the cells that were imaged were expressed with the respective GFP-TACC3 proteins (shown in first image in each captured at t = 0, GFP is displayed in grey colour). The respective time-lapse movies are shown in Additional file 4: Movie S4, Additional file 5: Movie S5. **e***.* The plot shows total mitotic progression time in WT vs. S558A mutant condition. Data are mean +/− S. E. (three experiments)

To further identify any loss of function defects induced by the phospho-deficient mutation, we imaged mitotic spindles by time-lapse microscopy in live α-tubulin-GFP- and H2B-mCherry- stably expressed HeLa Kyoto cells that were mitotic synchronized by thymidine and were imaged at the same stage (metaphase) of mitosis by following thymidine release (Methods). While in the TACC3 (WT)-expressed cells, the mitotic spindle apparatus appeared to be firmly positioned throughout the process of chromosome congression and segregation (Fig. 2Ai, Additional file 1: Movie S1); in the S558A mutant-expressed cells, the spindle-poles often failed to fix their positions stably along the pole-to-pole axis (Fig. 2Aii, Additional file 2: Movie S2). The whole spindle apparatus appeared to move and rotate randomly reflecting defects in localizing the spindle poles timely at correct positions. In the S558D TACC3-expressed cells, spindle-pole localization was normal like the WT cells, indicating that the phospho-mimicking form could rescue the TACC3 depletion-induced spindle pole-positioning defect (Fig. 2Aiii, Additional file 3: Movie S3). Since microtubules in these cells were also GFP-labeled, the expressions of GFP-TACC3 proteins in these cells were confirmed by Western blot (Fig. 2c). Detailed analysis of the time lapse movies revealed that the time for metaphase to anaphase transition was increased almost 70–80% as compared to the either TACC3 WT or S558D mutant cells (Fig. 2b). As astral microtubules play essential role in positioning the spindle poles during mitosis, the results imply that the defect is attributed to the loss of astral microtubules due to phospho-deficient mutation in TACC3. Spindle-pole positioning defects may reflect delays in total mitosis progression. To assess that, mitosis progression time was measured in the mutant cells as compared to the WT cells by live imaging. The cells were synchronized by thymidine treatment followed by release from thymidine prior to time-lapse imaging. It was also confirmed that the GFP-TACC3 WT or GFP-TACC3 S558A was transfected in the cells that were imaged for mitotic progression in the bright-field channel. Snapshot images of the GFP expressions of the same cells taken immediately before acquiring the bright-field time-lapse images are shown (Fig. 2d, on top and bottom of respective bright-field images). Mitosis progression was substantially delayed in the S558A mutant cells as compared to the WT cells (Fig. 2d; Additional file 4: Movie S4, Additional file 5: Movie S5). While the WT cells completed mitosis in ~ 45 min, S558A mutant cells took an average of 1 h 20 min to complete mitosis (plot in Fig. 2e).

### Ser558 phosphorylation does not alter TACC3-induced microtubule assembly in vitro

**Fig. 3:**
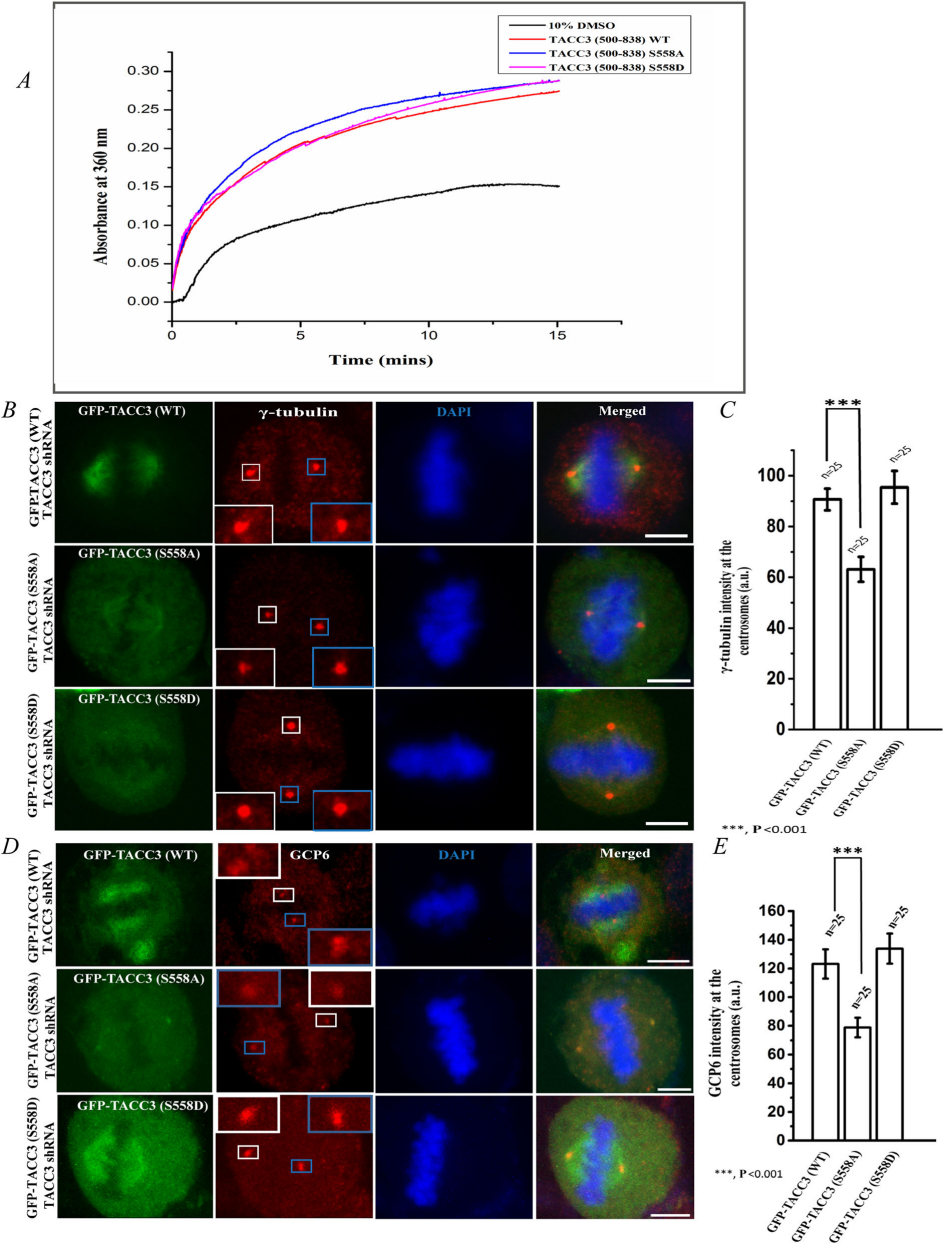
TACC3 S558A mutation affects recruitment of γ-TuRC proteins to the centrosomes. **a***.* The polymerization of αβ-tubulin (15 μM) in the presence of recombinant TACC3 (500–838) WT (red line), TACC3 (500–838) S558A (blue line), and TACC3 (500–838) S558D (pink line) was measured by turbidity assay. TACC3 proteins used were 15 μM, each. The curves represent the amount of turbidity resulting from microtubule polymerization under each condition. Polymerization curve of tubulin by 10% DMSO as polymerization inducer is also shown (black line). **b***.* Representative confocal images of GFP-TACC3 (WT)-TACC3 shRNA (top panel), GFP-TACC3 (S558A)-TACC3 shRNA (middle panel) or GFP-TACC3 (S558D)-TACC3 shRNA (bottom panel)-transfected (48 h) mitotic metaphase synchronized HeLa Kyoto cells showing the localization of γ-tubulin. Enlarged images of γ-tubulin localization are shown in insets. Scale bar, 5 μm. γ-tubulin (red) was stained with rabbit polyclonal anti-γ-tubulin antibody. **c***.* Quantification of intensities (sum of both poles) of γ-tubulin fluorescence at the centrosomes/spindle poles in GFP-TACC3 (WT)-TACC3 shRNA, GFP-TACC3 (S558A)-TACC3 shRNA, and GFP-TACC3 (S558D)-TACC3 shRNA transfected (48 h) metaphase cells. The bars represent mean +/− S.E. (three independent experiments). Statistical analysis between WT and S558A mutant data is shown. **d***.* Representative confocal images of GFP-TACC3 (WT)-TACC3 shRNA (top panel), GFP-TACC3 (S558A)-TACC3 shRNA (middle panel) or GFP-TACC3 (S558D)-TACC3 shRNA (bottom panel) transfected (48 h) mitotic metaphase synchronized HeLa Kyoto cells showing the localization of GCP6. Enlarged images of centrosomal GCP localization are shown in insets. Scale bar, 5 μm. GCP6 (red) was stained with rabbit polyclonal anti-GCP6 antibody. **e***.* Quantification of intensities (sum of both poles) of GCP6 fluorescence at the centrosomes/spindle poles in GFP-TACC3 (WT)-TACC3 shRNA, GFP-TACC3 (S558A)-TACC3 shRNA, and GFP-TACC3 (S558D)-TACC3 shRNA transfected (48 h) metaphase cells. The bars represent mean +/− S.E. (three independent experiments). n refers to number of cells analyzed

Since phospho-deficient mutation of Ser 558 of TACC3 induced loss of astral microtubules, we examined whether the phosphorylation exerts any direct role on microtubule assembly. Effect of Ser 558 phosphorylation of TACC3 on microtubule polymerization was assessed in vitro by using purified recombinant TACC3 proteins. Due to poor solubility, the full-length recombinant TACC3 could not be obtained. Instead, the domain (500–838) consisting of the TACC domain and the Ser 558 site; and its corresponding S558D or S558A mutant forms were purified. Either TACC3 S558A or TACC3 S558D mutant increased microtubule polymerization in vitro to a similar extent like the TACC3 WT (500–838) form (Fig. 3a), indicating that phosphorylation does not exert additional effect on microtubule polymerization.

### Phospho-deficient mutation of TACC3 affects integration of γ-TuRC proteins to the centrosomes

It has been demonstrated earlier that TACC3 interacts with the γ-TuRC proteins and is essential for integration of γ-TuRC to the centrosomes [9, 11]. Since Ser 558 phosphorylation itself does not affect microtubule polymerization, we then sought to investigate whether phosphorylation regulates γ-TuRC at the centrosomes, which could in turn cause loss of asters from the centrosomes. TACC3 (S558A)-GFP mutant was expressed in HeLa Kyoto cells with simultaneous depletion of endogenous TACC3 by shRNA. TACC3 (S558A) mutation resulted in significant loss of γ-tubulin from the centrosomes of mitotic synchronized metaphase cells (middle panel, Fig. 3b). In contrast, TACC3 (S558D) mutant could efficiently rescue the TACC3 depletion-induced loss of γ-tubulin from the centrosomes to a level that was nearly similar to TACC3-(WT)-expressed condition (Fig. 3b). Intensity analysis showed ~ 30% loss of γ-tubulin upon S558A mutation as compared either wild-type or S558D mutant condition (Fig. 3c). Similar defect was observed for the γ-TuRC protein, GCP6 at the centrosomes (Fig. 3d, e). Under similar condition, localization of centrosomal marker protein, pericentrin, was not affected (data not shown). Together, the results demonstrate that Ser 558 phosphorylation of TACC3 is essential for proper integration of the γ-TuRC proteins to the centrosomes.

### Ser 558-phosphorylated TACC3 interacts with γ-TuRC proteins

**Fig. 4:**
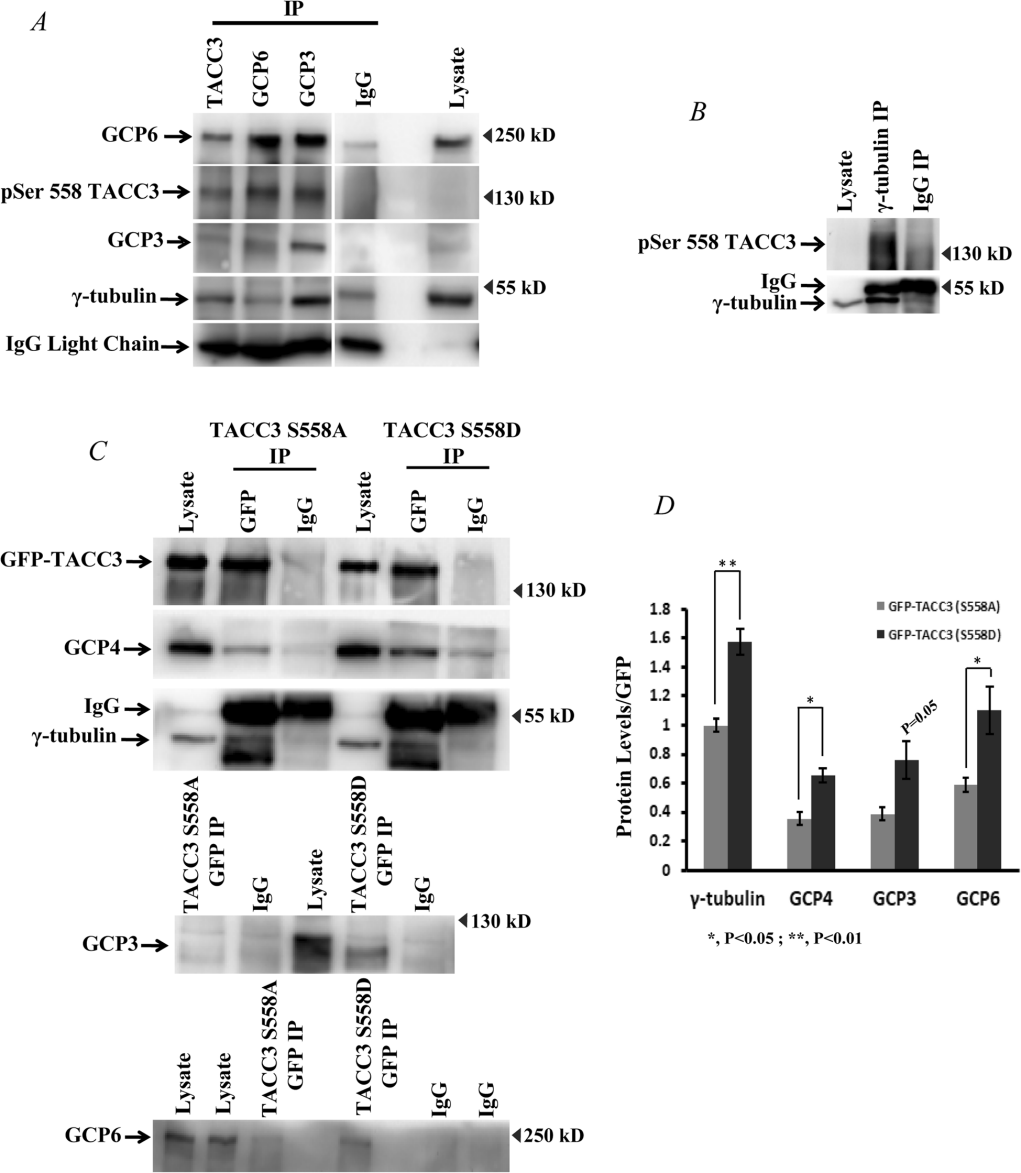
Ser558 phospho-TACC3 interacts with γ-TuRC. **a***.* Co-immunoprecipitation of TACC3, GCP3, GCP6 and **b***.* γ-tubulin from the lysates of HeLa cells. The immunoblot was probed for the presence of Ser 558 phosphorylated-TACC3 along with other ring complex proteins including γ-tubulin. **c***.* Co-immunoprecipitation of GFP-tagged TACC3 proteins using GFP antibody in the lysates of HeLa cells transfected with GFP-TACC3 (S558D)-TACC3 shRNA or GFP-TACC3 (S558A)-TACC3 shRNA. The immunoblots were probed for GFP-TACC3 S558D and GFP-TACC3 S558A form along with γ-tubulin, GCP4, GCP3, and GCP6 by using respective antibodies. **d***.* Fold change of different γ-TuRC proteins from the GFP-TACC3 immunoprecipitates of TACC3 S558A vs. S558D expressed cells are plotted (based on three experiments in each). Data are mean +/− S. E. Statistical analysis values are shown

As TACC3 phosphorylation is involved in stabilizing localization of γ-TuRC proteins to the centrosomes, we then examined whether the phosphorylated TACC3 mediates interaction with the proteins of γ-TuRC. Co-immunoprecipitation (co-IP) of GCP3, GCP6 and γ-tubulin in mitotic synchronized HeLa cell lysates followed by probing with phospho-site specific antibody of TACC3 revealed that the Ser 558-phosphorylated TACC3 remains associated with these proteins (Fig. 4a, b). The interactions were also confirmed by reverse-IP of TACC3 (Fig. 4a). We further assessed the effect of phospho-deficient Ser 558 site mutation on this interaction. For better comparison, we compared the interaction with that of the phospho-mimicking form. Co-IP with GFP antibody was performed with the lysates of mitotic synchronized HeLa cells expressed either with GFP-TACC3 S558A TACC3 shRNA or GFP-TACC3 S558D TACC3 shRNA. While the GFP-TACC3 S558D immono-precipitate showed considerable amount of γ-tubulin, GCP3, 4 and 6, respectively; the same was substantially reduced in the immuno-precipitate of GFP-TACC3 S558A mutant (Fig. 4c, d). Together, the results indicate that TACC3 phosphorylation at Ser 558 stabilizes TACC3-γ-TuRC interaction.

### Ser 558 phosphorylation of TACC3 is involved in stabilizing the assembly of γ-TuRC

**Fig. 5:**
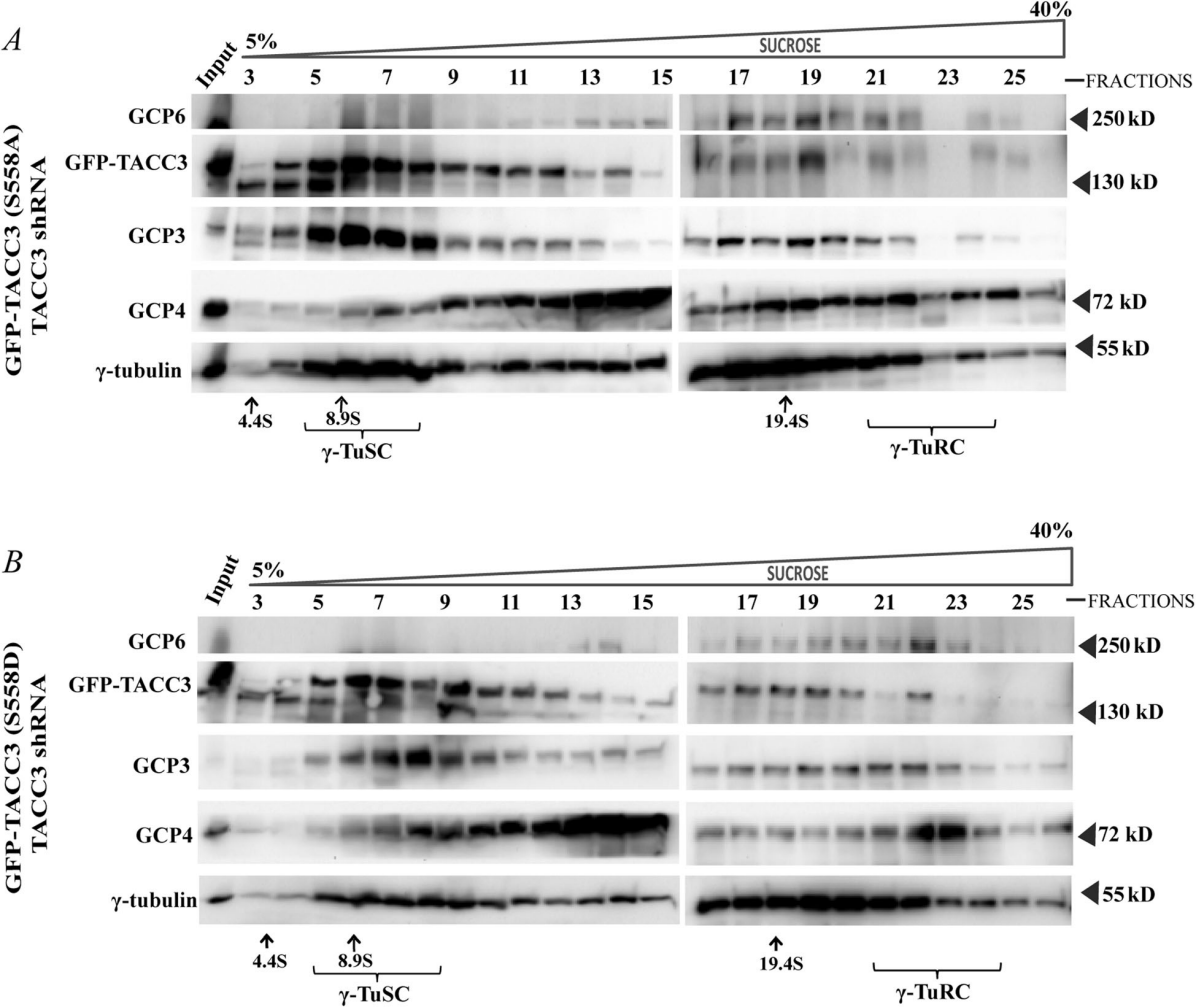
Phosphorylation of TACC3 at Ser558 is involved in the assembly of γ-TuRC from γ-TuSC. Sedimentation of γ-TuSC, and γ-TuRC along with GFP-TACC3 S558A or S558D from **a***.* GFP-TACC3KDP(S558A)-TACC3 shRNA vs. **b***.* GFP-TACC3KDP(S558D)-TACC3 shRNA transfected (48 h) HeLa cells. Cell lysates were centrifuged through 5–40% sucrose gradient at 150,000 X *g*, and the fractions collected from different layers of the gradient were then run through 10% SDS-PAGE followed by immunoblotting against γ-tubulin, GCP3, GCP4 and GFP-TACC3. Sedimentation coefficient (S) values of the protein complexes were assessed by calibrating against the known markers, BSA (66 kDa, 4.4 S), β-amylase (200 kDa, 8.9 S) and Thyroglobulin (669 kDa, 19.4 S) as shown. γ-TuSC and γ-TuRC were sedimented at 6–10 S and above 22 S, respectively

Since TACC3 phosphorylation at Ser 558 is involved in centrosomal recruitment of γ-TuRC proteins, we asked whether the phosphorylation plays any role in the stabilization of γ-TuRC complex assembly, specifically its assembly from the small complex components, γ-TuSCs. This was assessed by analyzing the relative abundance of the γ-TuRCs vs. γ-TuSCs in the lysates of HeLa Kyoto cells expressing GFP-TACC3(S558A)-TACC3 shRNA vs. GFP-TACC3 (S558D)-TACC3 shRNA by sucrose gradient-based sedimentation (Experimental Procedures). The levels of γ-TuRC only specific (sedimented at ~ 22 S) proteins, such as GCP4 and GCP6 were significantly higher in the lysates of GFP-TACC3 (S558D)-TACC3 shRNA expressed cells than those in the lysates of GFP-TACC3 (S558A)-TACC3 shRNA-expressed cells (Fig. 5 a, b). On the other hand, the levels of γ-tubulin small complex (γ-TuSC) (sedimented at a range of 6–10 S) was relatively more abundant in the lysates of GFP-TACC3 (S558A)-TACC3 shRNA-expressed cells than the GFP-TACC3 (S558D)-TACC3 shRNA-expressed cells. This implied that the assembly of γ-TuSC complexes onto γ-TuRC was impaired in the S558A mutant condition; whereas the assembly of γ-TuRC was stabilized in the S558D mutant condition. The results demonstrate that Ser 558 phosphorylated TACC3 plays an essential role in facilitating the assembly of the γ-tubulin ring complex from its components.

## Discussion

Aurora A plays crucial roles in centrosome function during mitosis as it regulates recruitment of a number of essential proteins to the peri-centriolar material (PCM) [16, 21, 27]. TACC3 is one such factor that is recruited to the PCM in a phosphorylation-dependent manner, specifically via Aurora A kinase [19, 22, 27]. However, the exact molecular role of the phosphorylated TACC3 at the PCM was not clearly understood. We have demonstrated here that TACC3 phosphorylation at the conserved Aurora A-targeting S558 site positively regulates the γ-tubulin ring complex assembly and its integration to the mitotic centrosomes. We have shown that in the Ser558 phosphorylated form; TACC3 associates with the γ-tubulin ring complex. The phosphorylation is critical for the interaction because mutation of the phosphorylation site to a non-phosphorylable amino acid significantly abolishes TACC3 association with the γ-tubulin ring complex (Figs. 4, 5). Such a role of the phospho-TACC3 in stabilizing the γ-tubulin ring complex is likely to have an impact on microtubule assembly and organization at the centrosomes since the γ-tubulin ring complex is the major nucleating machinery at the centrosomes. Supportively, our imaging data have shown that Ser 558 mutation to alanine in TACC3 results in loss of astral microtubules from the mitotic spindle poles in human cells. This phenotype is consistent with a previous study in *Drosophila*, where phosphorylation of an orthologous Ser residue in D-TACC was shown to exert loss of mitotic asters in the fly embryo [25]. In further support of the role of phosphorylation, specifically in human cell, we have shown that mutation of the Ser 558 site to a phospho-mimicking residue rescued the astral microtubules to an extent similar to the TACC3-WT- expressed cells (Fig. 1). Consistent with such an essential role in astral microtubule assembly, our study here additionally has identified characteristic defects in mitosis induced by the loss of phosphorylation at Ser 558 site. By live cell imaging, we have shown that mitotic cells expressed with the phospho-deficient TACC3 mutant exhibit defects in proper positioning of the centrosomes/spindle-poles. As a result, the cells take prolonged time to localize the spindle axis and therefore, the metaphase to anaphase progression is delayed. Proper connection of astral microtubules with the cell cortex is crucial for establishing correct positioning of the spindle poles. Therefore, the loss of the astral microtubules from the spindle poles is likely to be one of the key reasons behind the spindle-pole positioning defect. Live imaging of cells expressed with the pseudo-phosphorylated TACC3 form, which is able to rescue the astral microtubules and the spindle-pole positioning defect, further substantiates this possibility. In view of these findings, an obvious question arises though whether TACC3 phosphorylation has any direct role in astral microtubule formation. Our microtubule polymerization data have shown that though the coiled-coil TACC domain (600–838) on its own has a stimulating effect on microtubules assembly, which was reported earlier [9]; the phosphorylation as such does not exert any added effect on microtubule assembly (Fig. 3). This rules out the direct role of TACC3 phosphorylation in astral microtubule assembly and rather, supports the possibility that TACC3-phosphorylation-mediated stabilization of γ-tubulin ring complex can be responsible for the activation of astral microtubule assembly.

Earlier structural analyses indicated that the multiple γ-TuSC-consisting structures laterally associate with the sub-complexes of γ-tubulin with GCP4, GCP5 and GCP6 to complete the ring structure. The integration of those GCP4,5,6-containing γ-tubulin sub-complexes occurs at one specific side of the γ-TuRC wall [2, 3]. TACC3 has earlier been shown to stabilize integration of GCP4, GCP5 and GCP6 to the γ-TuRC and has been implicated to interlink GCP4/5/6 to γ-tubulin in the ring complex [9]. Results of the present study reveal that the Ser558 phosphorylation in TACC3 is critical for stabilizing TACC3 interaction with γ-tubulin, GCP4 and GCP6 (Fig. 4). Taken together, these observations suggest that Ser 558-phosphorylated TACC3 is involved in integrating γ-tubulin-GCP4/5/6 sub-complex to the γ-TuRC and to the centrosome.

How could the phospho-TACC3-mediated γ-tubulin ring complex stabilization regulate astral microtubules? Earlier studies in several model organisms including our work in human cells have demonstrated that TACC3 is required for astral microtubule assembly at the centrosomes, but is not indispensable for microtubule assembly via other nucleating sites [9, 17, 23, 24, 28, 29]. Additionally, in *Drosophila,* TACC3 phosphorylation by Aurora A has been shown to promote selectively the formation of mitotic asters. Mutation of D-TACC at Ser 863 leads to loss of asters without affecting spindle microtubules [18]. The previous findings in *Drosophila* and our present data in human cells together imply that the molecular mechanism regulating astral microtubule assembly is distinct than that of spindle microtubules. It is possible that a distinct pool of γ-tubulin ring complexes, such as the γ-tubulin-GCP4/5/6 sub-complexes, regulates assembly of the astral microtubules. TACC3 could organize and stabilize integration of those γ-tubulin sub-complexes in the ring complex in a manner that facilitates assembly of the astral microtubules and further, TACC3 phosphorylation can act as an “activation” signal for that process. Supporting such a role, we have shown that the phosphorylated TACC3 not only promotes centrosomal recruitment of the γ-tubulin ring complex proteins (Fig. 3); but is involved in stabilizing the ring complex assembly (Fig. 5). The interaction of TACC3 with the γ-tubulin complex proteins and the assembly of the ring complex from the small complexes, both were robustly affected, when the phosphorylation site was mutated to alanine (Figs. 4, 5). Although, we have shown association of the phosphorylated TACC3 with the γ-tubulin ring complex, but its bindings partner(s) in the ring complex remains to be determined in future. As TACC3 is intimately linked to cancer, it will also be interesting in future to investigate the possible link of TACC3-mediated γ-tubulin complex stabilization and astral microtubule assembly with tumorigenesis.

## Conclusions

We have uncovered in this work a hitherto unknown role of Aurora A site-specific TACC3 phosphorylation in regulation of the molecular integrity of the microtubule-nucleating protein complex, the γ-tubulin ring complex and its link to astral microtubule assembly at the centrosomes in human cells. As TACC3 shows frequent mutations in many cancers and induces cancer transformation, the results of this study may provide molecular basis of the cancer promoting activity of TACC3.

## Methods

### Reagents, and antibodies

DAPI was purchased from Sigma. Dulbecco’s modified Eagle’s medium (DMEM), fetal bovine serum (FBS), and antibiotic solutions were purchased from HiMedia, Inc. (Mumbai, India). Mouse monoclonal anti-TACC3, mouse polyclonal anti-GCP4, rabbit polyclonal anti-GCP6, and anti-γ-tubulin were obtained from Abcam; mouse monoclonal anti-α-tubulin and mouse monoclonal anti-γ-tubulin were purchased from Sigma; mouse monoclonal anti-GCP3, anti-GCP6 and goat polyclonal pericentrin antibody were obtained from Santa Cruz Biotechnology, Inc.; mouse monoclonal anti-actin was purchased from BD Biosciences; and rabbit polyclonal phospho-TACC3 antibody was purchased from Cell Signalling, USA. Rabbit polyclonal chTOG antibody was obtained from Abcam. The secondary antibodies, anti-mouse FITC, anti-rabbit TRITC, anti-mouse Cy5, anti-goat and anti-rabbit Cy5 were obtained from Jackson ImmunoResearch.

### Cell culture and transfection

HeLa Kyoto cells obtained from Daniel Gerlich, IMBA, Vienna, were cultured in DMEM containing 10% FBS at 37 °C under 5% CO2. GFP-TACC3 (WT)-TACC3shRNA GFP-TACC3 (S558A)-TACC3shRNA and GFP-TACC3 (S558D)-TACC3shRNA constructs, cloned in p-Brain plasmid were provided by Stephen J. Royle, University of Warwick, UK. These constructs were used to express the phospho-mutants of TACC3 and simultaneously deplete the endogenous TACC3 by TACC3 shRNA [26].

### Cell synchronization

For synchronization of cells at mitotic metaphase, the cells were treated with thymidine (2 mM) after 12 h of transfection by plasmids for 18 h and then released thereafter and were collected after 10 h of thymidine release, the time at which cells reached mitosis. The metaphase mitotic cells were confirmed based on the chromosome alignment at metaphase plate.

### Immunofluorescence microscopy

Cells fixed in methanol at − 20 °C were washed with phosphate-buffered saline (PBS), mixed with 1% bovine serum albumin and 0.1% Triton X-100, and subsequently incubated with primary antibodies for 2 h. After washing, the cells were then incubated with secondary antibodies and DAPI for 45 and 1 min, respectively. Coverslips were mounted using ProLong Gold anti-fade (Invitrogen), and images (63X) were captured using a Leica SP5 laser confocal microscope.

### Live cell imaging

All the time lapse imaging experiments were performed in HeLa Kyoto cells stably expressed with α-tubulin-GFP and H2B-mcherry and the cells were grown in glass bottom coverslip-coated chambers (Corning) at 37 °C. For capturing the spindle positioning of the mitotic cells, the cells after transfection with the plasmids (as specified) for 12 h, were treated with thymidine (2 mM) for 18 h and then thymidine was released to allow cell cycle progression to mitosis. Time lapsed image capturing of cells was started from the time (t = 0), when the chromosomes were visibly aligned to the metaphase plate and was continued till the end of mitosis (telophase). For measuring the total mitotic timing, the time-lapse bright-field images were captured at the entry (t = 0) of mitosis till telophase. Imaging was performed by Leica SP5 laser confocal microscope.

### Co-Immunoprecipitation (co-IP)

Cells were lysed with lysis buffer (4 °C) containing 20 mM Tris-HCl, pH 7.4, 0.1% Triton X-100, 50 mM NaCl, 1 mM EGTA, phosphatase inhibitors 2 and 3, and a protease inhibitor mixture (Sigma). γ-tubulin was immunoprecipitated using mouse monoclonal antibody followed by addition of protein G-agarose beads. The beads were washed with lysis buffer and then boiled in SDS-PAGE sample buffer for immunoblot analysis. Membranes were developed for immunoblot using the Immobilon reagent (Millipore), followed by imaging using ChemiDoc XRS System (Bio-Rad). Ser 558-phosphorylated TACC3 was probed by rabbit polyclonal phospho-TACC3 antibody (Cell Signaling, USA).

### Analysis of soluble γ-TuRCs by sucrose gradient

Cell fractionation was performed as described earlier [30] with slight modification. Briefly, cells were lysed with gradient buffer (50 mM HEPES, pH 7.4, 100 mM NaCl, 1 mM MgCl2 and 1 mM EGTA) supplemented with phosphatase inhibitors 2 and 3, and protease inhibitor. Then the lysate was overlaid onto the gradients of 5–40% sucrose in gradient buffer and fractionated by centrifugation at 50000 rpm for 5 h in a Beckman SW60Ti rotor. The fractions (0.1 ml each) were collected from the top to the bottom of the gradient. The proteins present in the fractions were then precipitated by cold ethanol and the precipitates were re-suspended in SDS-PAGE sample buffer prior to run in SDS-PAGE followed by Western blot. Markers with known sedimentation coefficients (S), BSA (66 kDa, 4.4 S), β-amylase (200 kDa, 8.9 S) and Thyroglobulin (669 kDa, 19.4 S) were run under similar condition to determine the position of unknown proteins or their complexes fractionated from the gradient.

### Protein purification

TACC3 (500–838) domain of WT, S558A and S558D of TACC3 were generated from human TACC3 cDNA (Origene, U.S.A.) by PCR and then sub-cloned into the pET15b vector, containing an N-terminal 6X-His tag; the resulting plasmids were transformed in BL21 (DE3) cells and the cells were grown at 16 °C under IPTG induction. The proteins were purified from the lysed cells through Ni^2+^-NTA column. αβ-tubulin was purified from goat brains through cycles of assembly and disassembly in vitro [31]. The resultant protein concentrations were estimated using the Bradford assay, with BSA as a standard [32].

### In vitro microtubule assembly

The time-dependent microtubule polymerization of αβ-tubulin (10 μM) in the absence and presence of purified recombinant TACC3 (500–838) WT vs. purified recombinant mutant proteins (10 μM each) was assessed at 37 °C by measuring the turbidity at 360 nm using a Varian Cary 50 BIO UV VIS spectrophotometer [33]. Briefly, the His-tagged TACC3 (500–838) WT, or His-TACC3 (500–838) S558A or His-TACC3 (500–838) S558D proteins were mixed with αβ-tubulin in PEM buffer (50 mM PIPES, 1 mM EGTA and 1 mM MgCl2, pH 6.8) and then GTP (1 mM) was added to the mixtures to induce microtubule polymerization. Microtubule polymerization of only αβ-tubulin (control) in the absence of any TACC3 proteins was induced by 10% DMSO.

### Image analysis

All immunofluorescence images were captured using a 63X (1.4 N.A.) oil immersion objective of a Leica SP5 confocal microscope. The same image acquisition and analysis settings were used for both control and treated conditions. The maximum intensity images were produced by projecting the images (Z-stacks) captured in three-dimensional optical sections at 0.25-μm intervals. The images were analyzed using Leica Application Suite Advanced Fluorescence Lite 2.8.0 software. Intensities were analyzed using Leica Application Suite Advanced Fluorescence Lite software. To analyze the intensities of *γ*-tubulin and GCP6 at the spindle poles (as shown in Fig. 3), the total intensity within an area spanning 15 μm^2^ and 1.7 μm^2^, respectively around the spindle poles (sum of intensity at two poles) was quantified in each case.

### Statistical analysis

The normality of the data was assessed using the Shapiro-Wilk test. The normally distributed data were analyzed with Student’s t test at the 99% confidence level. All data analyses were performed using R software. The data were plotted using Origin 8 or GraphPad Prism 6 software. The figures were organized using Adobe Photoshop and Adobe Illustrator.

## Supplementary information

**Additional file 1: Movie S1.** Movies constructed from the time lapse images of live HeLa kyoto cells stably expressed with α-tubulin-GFP and H2B-mCherry, that were transfected with GFP-TACC3 (WT)-TACC3 shRNA.

**Additional file 2: Movie S2.** GFP-TACC3 (S558A)-TACC3 shRNA.

**Additional file 3: Movie S3.** GFP-TACC3 (S558D)-TACC3 shRNA. The time t = 0 min refers to the time when the chromosomes were aligned to the metaphase plate based on H2B-mCherry staining.

**Additional file 4: Movie S4.** Movies constructed from the time lapse images of live HeLa kyoto cells stably expressed with α-tubulin-GFP and H2B-mcherry transfected with GFP-TACC3 (WT)-TACC3 shRNA.

**Additional file 5: Movie S5.** GFP-TACC3 (S558A)-TACC3 shRNA.

## Data Availability

All the data and materials are provided in the main paper and the additional files.

## Abbreviations

DAPI: 4′,6-diamidino-2-phenylindole
DMEM: Dulbecco’s Modified Eagle Medium
EGTA: Ethylene glycol-bis(β-aminoethyl ether)-N, N, N′, N′ tetraacetic acid
FBS: Fetal bovine serum
FITC: Fluorescein isothiocyanate
GCP: Gamma-tubulin complex protein
GTP: Guanosine triphosphate
PIPES: Piperazine-N,N′-bis (2-ethanesulfonic acid)
TRITC: Tetramethylrhodamine 5,6-isothiocyanate
γ-TuRC: Gamma-tubulin complex
